# Impact of Eggshell-Derived Calcium Oxide on Protein Cross-Linking and Gel Properties of Giant Snakehead (*Channa micropeltes*) Surimi

**DOI:** 10.3390/gels11030182

**Published:** 2025-03-06

**Authors:** Nattaporn Sanboonmee, Kriangsak Bunlue, Apipong Putkham, Hua Li, Sirithon Siriamornpun

**Affiliations:** 1 Department of Food Technology and Nutrition, Faculty of Technology, Mahasarakham University, Maha Sarakham 44150, Thailand; 65010853003@msu.ac.th (N.S.); kriangsak.b@msu.ac.th (K.B.); 2 Division of Environmental Technology, Faculty of Environment and Resource Studies, Mahasarakham University, Maha Sarakham 44150, Thailand; apipong.p@msu.ac.th; 3 Department of Cuisine and Nutrition, Yangzhou University, Yangzhou 225127, China; lihua216@yzu.edu.cn; 4 Research Unit of Thai Food Innovation (TFI), Mahasarakham University, Kantarawichai, Maha Sarakham 44150, Thailand

**Keywords:** surimi gelation, microstructure, rheology, disulfide bond, sulfhydryl groups

## Abstract

This study investigated the effects of calcium oxide (CaO) derived from eggshells on the gelation properties of surimi prepared from giant snakehead (*Channa micropeltes*). Surimi gels were enriched with CaO at concentrations of 0, 2, 4, 6, 8, and 10 µmol/100 g, and their physicochemical, rheological, and structural characteristics were evaluated. The optimal CaO concentration (6 µmol/100 g) significantly enhanced gel strength by 48.2%, breaking force by 26%, and deformation by 18% compared to the control (*p* < 0.05). Expressible moisture content decreased from 16.88% to 7.12%, while total sulfhydryl groups were reduced to 5.17 µmol/100 g. Rheological analysis revealed increased storage modulus (G′) and loss modulus (G″), indicating enhanced gel elasticity and viscosity during thermal processing. Scanning electron microscopy (SEM) demonstrated the formation of a compact, uniform gel network with fine pores at the optimal CaO concentration. SDS-PAGE analysis confirmed that CaO promoted transglutaminase (TGase) activity and TGase catalyzes the formation of cross-links between myosin heavy chain (MHC) and disulfide bonds. These results demonstrate the potential of eggshell-derived CaO as a sustainable, cost-effective additive to enhance surimi gel quality.

## 1. Introduction

Surimi, a processed fish protein product, is valued for its high protein content, low fat, and low cholesterol, making it a versatile ingredient in foods like kamaboko, fish tofu, fish balls, and imitation crab [1]. Its gelation, driven by myofibrillar proteins like myosin and actin, is critical for achieving desirable textural and functional properties, including elasticity, water retention, and gel strength. Proper heating during surimi processing, particularly at 40 °C, activates transglutaminase (TGase), an enzyme that forms a stable three-dimensional protein network. Subsequent heating at 80–90 °C ensures gelation, enhancing structural integrity. Optimizing surimi gelation is essential for producing high-quality products [2].

The giant snakehead (*Channa micropeltes*) is native to many tropical and subtropical regions. It is a freshwater, air-breathing, carnivorous fish that serves as an important source of protein worldwide [3]. The giant snakehead fish is an excellent candidate for surimi production due to its tender white flesh, minimal bones, and high protein content. These attributes make it a valuable raw material for creating high-quality surimi-based products. Due to its high nutritional value (high-quality protein and low fat) and pleasant flavor, surimi products are popular and consumed worldwide [4]. However, improving the gelation properties of giant snakehead surimi remains a challenge requiring further exploration.

Calcium ions (Ca^2+^), classified as denatured salts, reduce the free energy needed to transfer non-polar groups into water, facilitating protein unfolding and cross-linking [5]. Ca^2+^ induces the unfolding of fish myosin, exposing active functional groups. Additionally, Ca^2+^ disrupts the α-helical structure of myosin, enhancing hydrophobic interactions and textural properties [6]. Moreover, endogenous TGase, a Ca^2+^-dependent enzyme, can be activated by adding calcium compounds to surimi paste, thereby enhancing the texture of surimi gels [7,8].

Eggshells, a renewable by-product of the food industry, are a cost-effective source of calcium oxide (CaO), which can be obtained through calcination at 800 ± 1 °C, yielding over 98% purity [9]. CaO has shown potential for enhancing gelation, elasticity, and structural integrity in food products. It promotes protein cross-linking through hydrophobic interactions and disulfide bond formation, strengthening the gel network [10]. The growing emphasis on sustainability has led to increasing interest in utilizing natural waste materials to develop high-performance functional additives. For instance, recent studies have explored the transformation of cellulose-based nanofibers into carbon nanofibers (CNFs) with exceptional properties, demonstrating their potential applications in electronics, energy storage, and composite reinforcement [11]. Similarly, the valorization of eggshell waste for food applications aligns with this sustainability-driven approach, offering an innovative solution for improving surimi gelation while promoting environmental responsibility. Although previous studies have investigated the use of calcium compounds in surimi gelation, the potential of eggshell-derived calcium oxide (CaO) as a sustainable and functional additive remains underexplored. Unlike conventional calcium compounds, eggshell-derived CaO offers a cost-effective, environmentally friendly alternative with promising applications in food processing.

This study aimed to evaluate the effects of eggshell-derived CaO on the gelation properties of giant snakehead surimi, focusing on its role in enhancing protein cross-linking and gel network formation, particularly through its interaction with myosin heavy chain (MHC) and disulfide bond formation. Additionally, the study sought to investigate the impact of CaO on improving gel strength, elasticity, and water-holding capacity, which are crucial for high-quality surimi-based products. Microstructural characteristics were also analyzed using scanning electron microscopy (SEM) to provide insights into the effect of CaO on surimi gel formation. Furthermore, the study assessed potential limitations of CaO, including its influence on sensory attributes and long-term storage stability, in order to evaluate its practical applications in food processing.

## 2. Results and Discussion

### 2.1. Gel-Forming Properties

**Figure 1:**
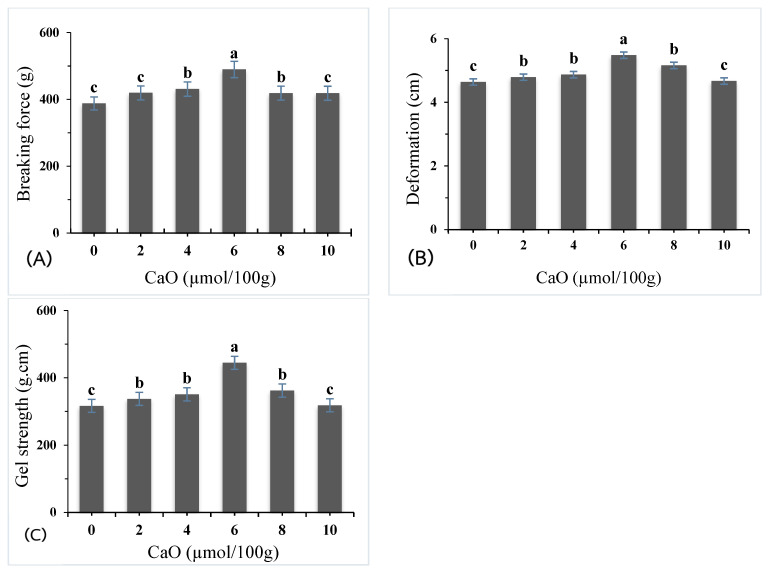
Breaking force (**A**), deformation (**B**), and gel strength (**C**) of surimi gel as affected by CaO at different concentrations. Different letters denote the significant difference (*p* < 0.05).

The gel properties of giant snakehead surimi supplemented with calcium oxide (CaO) derived from eggshells were evaluated at varying concentrations (0, 2, 4, 6, 8, and 10 µmol/100 g). The breaking force of the surimi gels significantly increased (*p* < 0.05) with CaO concentration, as shown in (Figure 1A) Interestingly, the breaking force increased markedly at 2 µmol/100 g, reaching its peak at 6 µmol/100 g, with a 26% improvement compared to the control. However, a significant decline in breaking force was observed at higher CaO concentrations (8–10 µmol/100 g), suggesting that excessive CaO compromises the gel’s structure.

Deformation values (Figure 1B) also demonstrated a significant improvement compared to the control, which exhibited a deformation value of 0.77 cm. At CaO concentrations of 2, 4, 6, 8, and 10 µmol/100 g, the deformation values were 0.80, 0.81, 0.91, 0.86, and 0.78 cm, respectively. The maximum deformation was achieved at 6 µmol/100 g, highlighting the improved elasticity and cohesive strength of the gel at this concentration. Beyond 6 µmol/100 g, the deformation values decreased with higher CaO concentrations.

Gel strength, a pivotal indicator of gel quality (Figure 1C), demonstrated a significant increase in all samples treated with calcium oxide (CaO) compared to the control sample (*p* < 0.05). The control gel exhibited a gel strength of 300.01 g·cm, while samples treated with 2, 4, 6, 8, and 10 µmol/100 g of CaO achieved gel strength values of 337.08, 350.61, 444.65, 361.97, and 327.84 g·cm, respectively. Remarkably, the greatest gel strength was observed at a CaO concentration of 6 µmol/100 g, representing a 48% enhancement over the control. The significant increase in gel strength after heat treatment is primarily due to the action of endogenous transglutaminase (TGase). TGase catalyzes the formation of ε-(γ-glutamyl) lysine cross-links between actomyosin molecules, improving the gel’s structural integrity and texture [7,8]. Furthermore, calcium ions were found to promote secondary cross-linking of protein chains, resulting in improved stability and texture properties [10]. The hydrophobic properties of calcium reduce intramolecular hydrogen bonding and hydrophobic interactions, further enhancing gel stability. Calcium also integrates into the gel matrix, supporting stronger gelation [12].

At higher CaO concentrations (8–10 µmol/100 g), gel-forming properties slightly declined, likely due to reduced myosin heavy chain (MHC) cross-linking. Excessive Ca^2+^ can disrupt optimal gel network formation during heating [6]. Elevated Ca^2+^ levels may form salt bridges between Ca^2+^ and carboxyl groups of amino acids or protein C-terminals, leading to excessive denaturation, precipitation, and the formation of large protein complexes [13]. These high Ca^2+^ concentrations inhibit MHC cross-linking, reducing gel formation and solubility. Calcium ions at elevated levels create protein–Ca–protein bridges, resulting in a firmer but less elastic gel [14]. Higher CaO concentrations increase Ca^2+^ availability, strengthening ionic interactions that promote protein aggregation. Excessive Ca^2+^ may also lead to larger colloidal particle formation, disrupting the gel network and weakening overall gel strength [15].

### 2.2. Expressible Moisture Content, Whiteness, TCA-Soluble Protein, and Total Sulfhydryl Groups

As shown in Table 1, the incorporation of calcium oxide (CaO) into surimi gels significantly enhanced their physicochemical properties, including expressible moisture, whiteness, TCA-soluble protein, and sulfhydryl groups, underscoring its effectiveness in improving gel quality.

The addition of CaO markedly reduced the expressible moisture content compared to the control sample (*p* < 0.05), with the lowest value observed at 6 µmol/100 g (7.12%), which is significantly lower than the value of the control (16.88%). This reduction indicates enhanced water-holding capacity (WHC), attributed to the alignment and water-binding capacity of myosin proteins during the heating process. The stability of the protein network, facilitated by Ca^2+^ ions binding to negatively charged myofibrils and forming protein–Ca^2+^–protein cross-links, further contributes to improved WHC [16].

CaO also significantly influenced the whiteness of giant snakehead surimi gels (*p* < 0.05). Whiteness values progressively increased with CaO concentration, peaking at 6 µmol/100 g (71.49) compared to the control (69.04). This enhancement is likely due to the strengthening of the gel’s structural integrity, uniform protein aggregation, and reduced discoloration during heating. Improved gel strength further contributes to a denser texture and more uniform protein distribution, reducing light scattering and resulting in greater whiteness [17,18].

In addition, CaO significantly reduced TCA-soluble protein content (*p* < 0.05), indicating decreased protease activity and enhanced protein stability. Gels treated with intermediate CaO concentrations (2–6 µmol/100 g) exhibited lower TCA-soluble protein levels compared to the control and 10 µmol/100 g CaO, reflecting improved protein cross-linking. These results align with studies demonstrating that Ca^2+^ enhances MHC cross-linking and gel strength while minimizing proteolytic degradation [7,8]. Furthermore, calcium inhibits protease activity, reduces protein breakdown, and promotes stable gel networks, leading to improved water retention and gel stability [19].

The addition of CaO significantly reduced the total sulfhydryl content (*p* < 0.05). The control gel contained 13.12 µmol/g of sulfhydryl groups, while CaO-treated gels (2–10 µmol/100 g) showed significantly lower levels (4.48–5.80 µmol/g). This reduction indicates enhanced disulfide bond formation, resulting from myosin heavy chain (MHC) polymerization through sulfhydryl oxidation during mixing and heating, facilitated by increased oxidizing agents [20]. In summary, the addition of CaO remarkably enhanced gel properties by improving water-holding capacity (WHC), whiteness, protein cross-linking, and disulfide bond formation. CaO helps develop a stable, high-quality gel network, making it a valuable additive for surimi products.

### 2.3. Rheological Properties

**Figure 2:**
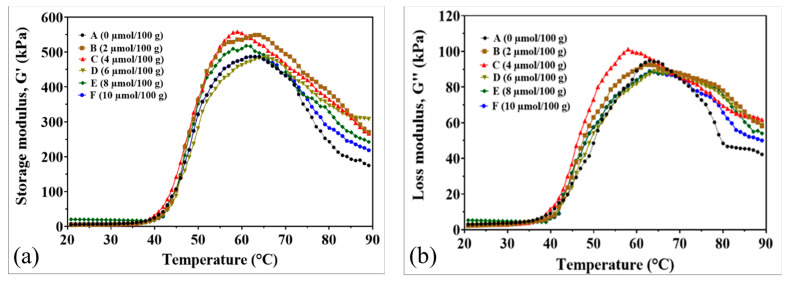
Rheological properties of surimi gel affected by CaO at different concentrations. (**a**) Storage modulus G′. (**b**) Loss modulus G″. (A) Control, 0 µmol/100 g. (B) 2 µmol/100 g. (C) 4 µmol/100 g. (D) 6 µmol/100 g. (E) 8 µmol/100 g. (F) 10 µmol/100 g.

The dynamic rheological properties of surimi gels supplemented with CaO showed significant improvements in gel strength, elasticity, and viscosity, as demonstrated by the storage modulus (G′) and loss modulus (G″) analyses (Figure 2). The storage modulus (G′), indicating gel elasticity and network formation, increased with rising CaO concentrations during heating from 40 to 65 °C (Figure 2a). Gels treated with 2–10 µmol/100 g CaO exhibited higher G′ values than the control, suggesting that CaO enhances myosin gelation. This enhancement is due to the exposure of hydrophobic groups in myosin at elevated temperatures, promoting disulfide bond cross-linking and hydrophobic interactions between protein molecules [21,22]. These interactions lead to protein aggregation and the formation of an irreversible, cross-linked network, strengthening the gel matrix and increasing elasticity [23]. Additionally, CaO stimulated transglutaminase (TGase) activity, facilitating the formation of ε-(γ-glutamyl)-lysine bonds, which further improved the protein cross-linking, mechanical properties, and water-holding capacity (WHC) of the surimi gels [24].

The loss modulus (G″), indicating the gel’s viscous behavior during heating, also increased with CaO addition, particularly between 40 and 63 °C (Figure 2b). The highest G″ value was observed at 4 µmol/100 g CaO, suggesting optimal protein interactions and hydration at this concentration. Beyond 65 °C, G″ values declined as the gel transitioned from a semi-fluid to a solid structure, with higher CaO concentrations further reducing viscosity due to enhanced protein cross-linking and gel stabilization.

The supplementation of CaO significantly enhanced the structural and functional properties of the surimi gel matrix by facilitating covalent bond formation and promoting myosin cross-linking. This process resulted in a gel with increased strength, stability, and water-holding capacity, suggesting that the release of Ca^2+^ ions from CaO further strengthened the gel network by catalyzing TGase activity and supporting ε-(γ-glutamyl)-lysine bond formation, thereby enhancing the gel’s mechanical properties and elasticity [6,25]. Our current study primarily focused on temperature-dependent rheological properties (G′ and G″), but we recognize that frequency sweep tests would further enhance the characterization of CaO’s effects on gel behavior.

### 2.4. Scanning Electron Microscopy (SEM)

**Figure 3:**
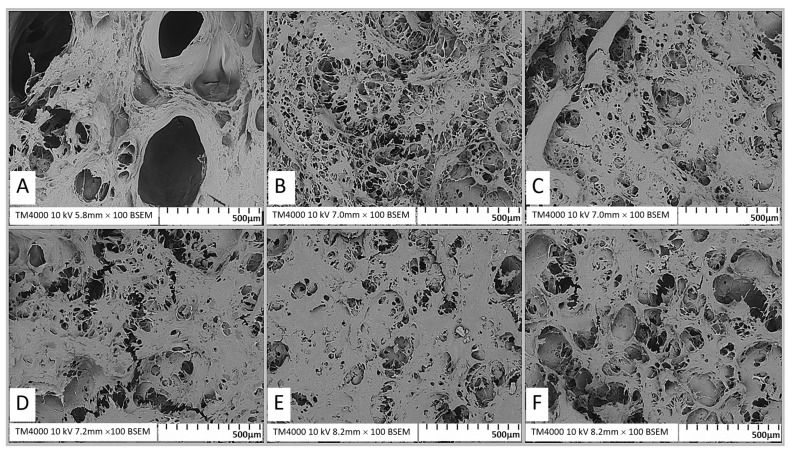
Scanning electron microscopy (SEM) images of surimi gel affected by CaO at different concentrations. Images were captured at ×100. (**A**) Control, 0 µmol/100 g; control; (**B**) 2 µmol/100 g; (**C**) 4 µmol/100 g; (**D**) 6 µmol/100 g; (**E**) 8 µmol/100 g; (**F**) 10 µmol/100 g.

The microstructure of protein gels is a key determinant of their functional and textural properties. Scanning electron microscopy (SEM) analysis of surimi gels treated with varying concentrations of calcium oxide (CaO) revealed distinct structural differences (Figure 3). The control gel (0 µmol/100 g) exhibited large air voids and a loose network with substantial internal cavities, indicating incomplete protein aggregation (Figure 3A).

With the addition of CaO, the gel’s structure improved significantly. At 2–4 µmol/100 g, the network became denser, with smaller, evenly distributed pores and a more uniform structure (Figure 3B,C). These changes are attributed to enhanced hydrophobic interactions and disulfide bond formation during heat treatment, which strengthened the gel’s matrix and improved its water-holding capacity [26,27,28]. At 6–8 µmol/100 g (Figure 3D,E), the gels exhibited a compact, highly uniform network with fine pores, reflecting optimal protein cross-linking and aggregation. This denser structure enhanced gel strength and water retention [28,29]. Stronger gels with more homogeneous networks also correlated with improved visual properties [18]. However, at 10 µmol/100 g (Figure 3F), the gel’s network deteriorated, showing larger holes and thicker protein filaments. This loose structure, caused by excessive protein aggregation, reduced gel strength and stability [30].

These findings highlight the role of CaO in enhancing protein cross-linking and gel structure, leading to improved functional properties. The observed microstructural changes align with enhanced gel strength and water retention, supporting CaO as a valuable additive for surimi gel production.

### 2.5. Sodium Dodecyl Sulphate- Polyacrylamide Gel Electrophoresis (SDS-PAGE)

**Figure 4:**
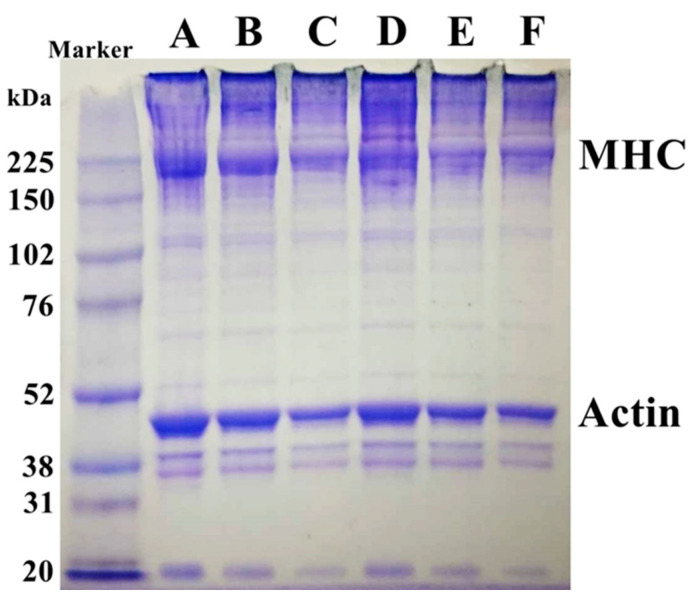
SDS-PAGE patterns of surimi gels affected by CaO at different concentrations. (A) Control, 0 µmol/100 g; (B) 2 µmol/100 g; (C) 4 µmol/100 g; (D) 6 µmol/100 g; (E) 8 µmol/100 g; (F) 10 µmol/100 g.

Changes in the protein patterns of surimi gels from giant snakehead fish with varying calcium oxide (CaO) concentrations derived from eggshells were examined through SDS-PAGE analysis, as shown in Figure 4. The control sample (0 µmol/100 g) showed distinct bands for MHC and actin, key structural proteins in surimi gel formation. As the CaO concentration increased from 2 to 10 µmol/100 g, a reduction in the intensity of the MHC and actin bands was observed. Additionally, higher molecular weight protein bands above the MHC band indicated myosin polymerization into larger aggregates. This is attributed to the activation of transglutaminase (TGase), which facilitates MHC cross-linking into higher molecular weight polymers, enhancing the gel’s network and texture [8].

These findings suggest that the degree of cross-linking is positively correlated with gel strength. As the treatment conditions become more complex, the increased myosin cross-linking strengthens the gel’s structure [18]. Darker bands at the wells were observed, likely due to minor protein aggregation or incomplete solubilization. However, this did not affect the overall interpretation of protein cross-linking and myosin polymerization trends, as key protein bands remained clearly distinguishable. Standardized sample preparation minimized potential inconsistencies. The results confirm that CaO incorporation strengthens the gel by promoting both disulfide and non-disulfide covalent bonds, ultimately improving surimi gel quality. While CaO has been shown to enhance gel strength and structural integrity, its impact on sensory attributes, such as taste and odor, requires further investigation. High CaO concentrations may introduce a slightly alkaline taste, which could affect overall product acceptability. Additionally, its influence on long-term storage stability remains unclear, particularly in terms of potential changes in texture and moisture retention over extended storage periods.

## 3. Conclusions

This study demonstrates that eggshell-derived calcium oxide (CaO) enhances the gelation properties of giant snakehead (*Channa micropeltes*) surimi. The optimal CaO concentration (6 µmol/100 g) significantly improved gel strength, deformation, and water-holding capacity while promoting protein cross-linking and a more compact microstructure. These benefits were achieved without affecting protease activity or gel whiteness, highlighting the potential of using CaO as a sustainable and cost-effective additive for surimi quality enhancement. However, excessive CaO may negatively impact gel texture and elasticity due to protein over-aggregation. Its potential effects on sensory attributes, long-term stability, and consumer acceptance require further investigation. Future research should also assess its interactions with transglutaminase, its freeze–thaw stability, and safety aspects to ensure regulatory compliance. Addressing these factors will help optimize the use of eggshell-derived CaO in surimi production while supporting sustainable waste management.

## 4. Materials and Methods

### 4.1. Surimi Preparation

Giant snakehead was purchased from a market in Kantharawichai district, Mahasarakham Province, Thailand. The fish was beheaded and gutted, and scales were removed. The flesh was separated from the bones, and the fish meat was minced using a meat grinder. The fish mince was washed with a cold 0.1% NaCl solution (4 ± 1 °C) at a 2:1 water to mince ratio (v/w). After 3 min of stirring, the wash was removed using cheesecloth for manual dewatering. The surimi was then mixed with 4% sucrose, 4% sorbitol as cryoprotectants, and 0.1% sodium tripolyphosphate, packed into polyethylene bags (200 g), frozen, and stored at −20 ± 1 °C until use.

### 4.2. Calcium Oxide (CaO) Preparation

Calcium oxide (CaO) was prepared from hatchery eggshell waste following the methods described by Chuakham et al. and Putkham et al. [9,31]. The eggshells were thoroughly washed and sun-dried to remove residual membranes and then ground and sieved to a particle size of 250 µm. The processed material was subsequently subjected to calcination in a laboratory rotary kiln at 800 ± 1 °C for 1 h, operating at a rotation speed of 1 rpm under an air atmosphere. The physical and chemical characteristics of the bio-CaO were systematically analyzed using advanced techniques, including scanning electron microscopy with energy-dispersive spectroscopy (SEM-EDS), powder X-ray diffraction (PXRD), X-ray fluorescence spectrometry (XRF), and atomic absorption spectroscopy (AAS). The resulting bio-calcined product contained approximately 98% nano-calcium oxide, with a mean crystallite size of 47.5 nm, exhibiting properties comparable to commercial calcium carbonate. This preparation method is cost-effective, environmentally sustainable, and highly efficient. Moreover, the bio-derived CaO meets food-grade specifications established by the Joint FAO/WHO Expert Committee on Food Additives (JECFA), the European Union (EU), and Thailand’s Food and Drug Administration.

### 4.3. Surimi Gel Preparation

The frozen giant snake head surimi was cut into small pieces after thawing at 4 ± 1 °C to equilibrate at room temperature. The thawed surimi was minced for 1 min using a food processor (MK-5086M, Panasonic Co., Ltd., Shinagawa-ku, Japan). The minced surimi was then combined with 2.5% NaCl for 1 min, followed by another session of mixing with calcium oxide solution at varying concentrations (0, 2, 4, 6, 8, and 10 µM/100 g surimi). The mixture was prepared with a moisture content adjusted to 80%, followed by mixing under 10 ± 1 °C for 2 min. The resulting paste (actomyosin sol) was packed into 2.5 × 2.5 cm stainless steel molds, wrapped in polyethylene film, and set at 40 ± 1 °C for 30 min. The samples were then heated at 80 ± 1 °C for 20 min, followed by rapid cooling in an ice bath for 10 min. The resulting gels were stored overnight at 5 °C before evaluating their gel-forming properties.

### 4.4. Determination of Gel Strength

The gel-forming ability was evaluated in terms of breaking force and deformation using a Texture Analyzer TA-XT2i (Stable Micro Systems, Godalming, UK) equipped with a spherical probe (P/5S) at a test speed of 1 mm/s. Breaking force was defined as the maximum force required to penetrate the sample’s surface, while deformation referred to the distance travelled by the probe from initial contact to full penetration. Gel strength was calculated as the product of breaking force (g) and deformation (cm).

### 4.5. Determination of Gel Whiteness

The whiteness of surimi gel was evaluated following the method described by Xue et al. [32] using a Minolta CR-400 colorimeter, based on the CIE L* a* b* color system. In this system, L* represents lightness, a* denotes the red–green axis, and b* corresponds to the yellow–blue axis. The whiteness value was then calculated using the following equation:

Whiteness = 100 − [(100 − L*)^2^ + a*^2^ + b*^2^]^1/2^ (1)

### 4.6. Determination of Expressible Moisture Content

Expressible moisture content was determined following the method adapted from Huang et al. [12]. Samples were cut into 5 mm thick slices and initially weighed (X). The slices were placed between layers of filter paper, with three layers beneath and two layers above, and enclosed in a zip-lock bag to minimize evaporation during the pressing process. A standardized weight of 5 kg was applied for 2 min. After pressing, the samples were reweighed (Y). The expressible moisture content was calculated using the following equation:

Expressible moisture content (%) = 100 × [(X − Y)/X](2)

### 4.7. Determination of TCA-Soluble Protein

Three grams of giant snakehead surimi gel were combined with 27 mL of 5% trichloroacetic acid (TCA) solution, homogenized, and filtered through No. 4 filter paper. The filtrate volume was adjusted to 50 mL, and the clear supernatant was analyzed for soluble protein content following the method of Lowry et al. [33].

### 4.8. Determination of Total Sulfhydryl Groups

The total sulfhydryl content in surimi gels was quantified using Ellman’s method [34]. A 0.1 g gel sample was prepared in a pH 8 buffer containing 8 mol/L urea, 2% SDS, 10 mmol/L EDTA, and 0.1 mol/L phosphate (pH 7) and then homogenized at 1000 rpm for 5 min using a Teflon homogenizer (Schuett-Biotec Homogenizer, Calibre Scientific, UK). The mixture was incubated in the dark at 40 °C for 15 min before reacting with 5,5′-dithiobis-(2-nitrobenzoic acid) (DTNB). The absorbance at 412 nm was recorded, and the sulfhydryl concentration was calculated using DTNB’s molar extinction coefficient of 13,600 M^−1^ cm^−1^.

### 4.9. Dynamic Rheological Measurements

The dynamic rheological properties of surimi paste were assessed following the method of Lin et al. [35]. A rheometer (HAAKE MARS40, Thermo Fisher Scientific, Waltham, MA, USA) equipped with a P35/Ti rotor was used, with the platform temperature set to 20 °C. Measurements were conducted with a 1 mm gap, an oscillation frequency of 1.0 Hz, and a strain of 1.0%. A temperature sweep test was performed by gradually heating the samples from 20 ± 0.1 °C to 90 ± 0.1 °C at a rate of 2 ± 0.1 °C/min while continuously recording the storage modulus (G′) and loss modulus (G″) at one-minute intervals.

### 4.10. Microstructure Analyses

The microstructure of the surimi gel was analyzed using scanning electron microscopy (SEM) following the method by Huang et al. [12]. The surimi sample was freeze-dried for 12 h and then cut into 2 mm thick slices. The sample was gold-coated, and the microstructure was imaged using a Hitachi TM 4000 Plus scanning electron microscope (Hitachi, Chiyoda City, Japan). Sample images were captured at a magnification of ×100.

### 4.11. SDS-PAGE Analyses

Protein structural changes in surimi gel were examined using SDS-PAGE, following the method of Weber and Osborn [36], with slight modifications. A 0.1 g surimi gel sample was homogenized at 1000 rpm for 4 min in 8 mL of 0.1 mol/L phosphate buffer (pH 6.8) containing 8 mol/L of urea and 2% SDS. The homogenate was then boiled (100 ± 0.1 °C) for 2 min, rapidly cooled in ice water, and stored at 5 ± 0.1 °C overnight before preparing the reduced samples. For sample reduction, 2 mL of the boiled solution was mixed with 2 mL of a reagent containing 0.05% bromophenol blue, 50% glycerol, 0.4% SDS, 0.5 mol/L phosphate buffer (pH 6.8), and 30% 2-mercaptoethanol. Electrophoresis was performed using a 12% acrylamide separation gel and a 5% acrylamide stacking gel, with 10 μL per lane. The stacking gel was run at 80 V, followed by the separation gel at 120 V, using a PowerPac Basic system (Bio-Rad, Hercules, CA, USA). Proteins were visualized by staining with Coomassie Brilliant Blue R-250, followed by decolorization and imaging for further analysis.

### 4.12. Statistical Analysis

Statistical analysis was conducted using one-way analysis of variance (ANOVA), followed by Duncan’s multiple range test to determine significant differences among groups at a significance level of *p* < 0.05. Data were processed using SPSS for Windows version 17 (SPSS Inc., Chicago, IL, USA) and presented as mean ± standard deviation (SD).

## Figures and Tables

**Table 1:** Expressible moisture content, whiteness, TCA-soluble protein, and total sulfhydryl groups of surimi gel affected by CaO at different concentrations.

CaO (µmol/100 g)	Expressible Moisture Content (%)	Whiteness	TCA-Soluble Protein (mg/100 g)	Total Sulfhydryl Groups (µmol/g)
0	16.88 ± 0.87 ^a^	68.14 ± 0.86 ^c^	1.89 ± 0.26 ^a^	13.12 ± 0.11 ^a^
2	12.13 ± 0.42 ^b^	69.04 ± 0.63 ^b^	1.26 ± 0.18 ^c^	8.64 ± 0.08 ^b^
4	9.88 ± 1.81 ^c^	69.27 ± 0.61 ^b^	1.43 ± 0.12 ^c^	7.74 ± 0.14 ^c^
6	7.12 ± 1.31 ^d^	71.49 ± 0.20 ^a^	1.25 ± 0.07 ^c^	7.95 ± 0.10 ^c^
8	10.13 ± 0.24 ^bc^	71.01 ± 0.24 ^a^	1.31 ± 0.17 ^c^	7.86 ± 0.05 ^c^
10	11.07 ± 1.14 ^bc^	70.99 ± 0.74 ^a^	1.62 ± 0.09 ^b^	7.32 ± 0.13 ^d^

Note: Data were expressed as mean ± standard deviation. Different letters in the same column indicate significant differences among surimi gels (*p* < 0.05).

## Data Availability

Additional data are available upon request.
